# Diiodidobis[4-(4-nitro­benz­yl)pyridine-κ*N* ^1^]zinc

**DOI:** 10.1107/S1600536811005939

**Published:** 2011-02-23

**Authors:** Graham Smith, Urs D. Wermuth, Michael L. Williams

**Affiliations:** a Faculty of Science and Technology, Queensland University of Technology, GPO Box 2434, Brisbane, Queensland 4001, Australia; b School of Biomolecular and Physical Sciences, Griffith University, Nathan, Queensland 4111, Australia

## Abstract

The asymmetric unit of the title compound, [ZnI_2_(C_12_H_10_N_2_O_2_)_2_], obtained from the reaction of 4-(4-nitro­benz­yl)pyridine with zinc(II) iodide, contains two independent discrete distorted tetra­hedral complex units [Zn—I = 2.5472 (8)–2.5666 (7) Å and Zn—N = 2.044 (4)–2.052 (4) Å], which are essentially identical conformationally. The crystal used for measurement was a racemic twin.

## Related literature

For the structures of some Zn^II^–pyridine complexes, see: Le Querler *et al.* (1977 ▶); Pasaoglu *et al.* (2006 ▶); Fan & Wu (2006 ▶). For the structure of a mixed-ligand Pt^II^ complex with 4-(4-nitro­benz­yl)pyridine, see: Chan *et al.* (1993 ▶).
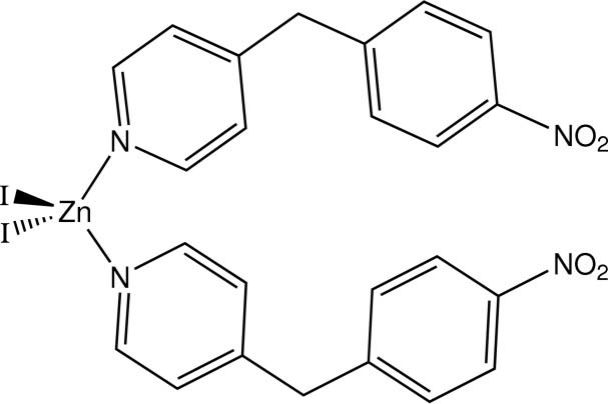


## Experimental

### 

#### Crystal data

[ZnI_2_(C_12_H_10_N_2_O_2_)_2_]
*M* *_r_* = 747.61
Orthorhombic, 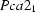

*a* = 18.2091 (2) Å
*b* = 15.8998 (3) Å
*c* = 19.0327 (3) Å
*V* = 5510.37 (15) Å^3^
*Z* = 8
Mo *K*α radiation
μ = 3.17 mm^−1^
*T* = 200 K
0.40 × 0.22 × 0.15 mm

#### Data collection

Oxford Diffraction Gemini-S Ultra CCD detector diffractometer
Absorption correction: multi-scan (*CrysAlis PRO*; Oxford Diffraction, 2010 ▶) *T*_min_ = 0.865, *T*_max_ = 0.980
18536 measured reflections
10102 independent reflections
7499 reflections with *I* > 2σ(*I*)
*R*_int_ = 0.024

#### Refinement

*R*[*F* ^2^ > 2σ(*F* ^2^)] = 0.032
*wR*(*F* ^2^) = 0.056
*S* = 0.89
10102 reflections
627 parameters
25 restraints
H-atom parameters not refined
Δρ_max_ = 0.63 e Å^−3^
Δρ_min_ = −0.47 e Å^−3^
Absolute structure: Flack (1983 ▶), 4516 Friedel pairs
Flack parameter: 0.430 (14)

### 

Data collection: *CrysAlis PRO* (Oxford Diffraction, 2010 ▶); cell refinement: *CrysAlis PRO*; data reduction: *CrysAlis PRO*; program(s) used to solve structure: *SHELXS97* (Sheldrick, 2008 ▶); program(s) used to refine structure: *SHELXL97* (Sheldrick, 2008 ▶) within *WinGX* (Farrugia, 1999 ▶); molecular graphics: *PLATON* (Spek, 2009 ▶); software used to prepare material for publication: *PLATON*.

## Supplementary Material

Crystal structure: contains datablocks global, I. DOI: 10.1107/S1600536811005939/ng5114sup1.cif

Structure factors: contains datablocks I. DOI: 10.1107/S1600536811005939/ng5114Isup2.hkl

Additional supplementary materials:  crystallographic information; 3D view; checkCIF report

## Figures and Tables

**Fig. 1.**
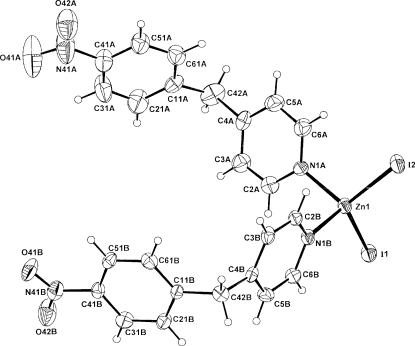
The molecular configuration and atom-numbering scheme for the first of the two independent complex units (about Zn1) in the asymmetric unit of (I), with non-H atoms drawn as 30% probability ellipsoids.

**Fig. 2.**
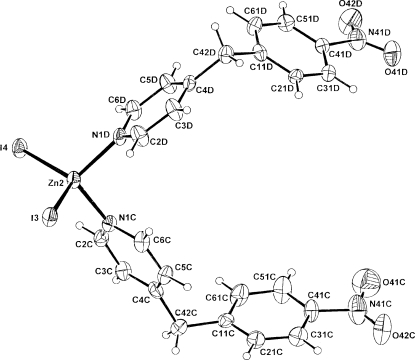
The molecular configuration and atom-numbering scheme for the second complex unit (about Zn2) in (I) (30% probability).

**Fig. 3.**
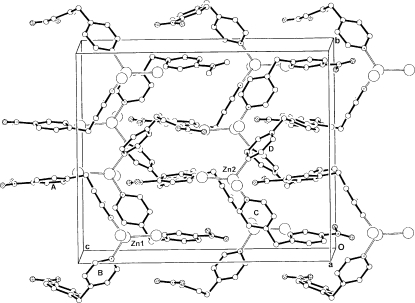
The packing of (I) in the unit cell, viewed down the a cell direction. Hydrogen atoms are omitted.

## supplementary crystallographic information

### Comment

The structures of complexes of ZnI_2_ with pyridine and substituted pyridine ligands, of the type [ZnI_2_(py)_2_] are common in the crystallographic literature *e.g.* with pyridine (Le Querler *et al.*, 1977), and with nicotinamide and isonicotinamide (Pasaoglu *et al.*, 2006). These complexes are usually discrete with distorted tetrahedral stereochemistry. Polymeric complexes having similar stereochemistry are also formed with bifunctional pyridines such as 4,4'-bipyridine (Fan & Wu, 2006). We obtained the title compound [ZnI_2_(C_12_H_10_N_2_O_2_)_2_] (I) from the reaction of zinc(II) iodide with 4-(4-nitrobenzyl)pyridine (*L*) and the structure is reported here. This substituted pyridine has only occasionally been used as a ligand in metal complex formation *e.g.* in the mixed-ligand Pt^II^ complex with 2,9-diphenyl-1,10-phenanthroline (Chan *et al.*, 1993).

In the structure of (I), the asymmetric unit contains two independent discrete distorted tetrahedral [ZnI_2_*L*_2_] complex units, involving Zn1 and Zn2 (Figs. 1, 2). The Zn—I range is 2.5472 (8)–2.5666 (7) Å, the Zn—N range is 2.044 (4)–2.052 (4) Å and the bond angle range about Zn is 98.99 (17)–119.96 (2)° (for I1—Zn1—I2 and N1*A*—Zn1—N1*B*, respectively). The two complex molecules are essentially identical conformationally and are related by pseudo-symmetry, being treated as a racemic twin in the structure refinement.

In the crystal packing of (I) there are only weak intermolecular aromatic *C*—*H*···O_nitro_ interactions [C5A—H···O41D, 3.211 (9) Å and C6B—H···O41B, 3.295 Å] but there are some aromatic ring π–π associations involving the C11B–C61B rings: ring centroid separation, 3.591 (3) Å; inter-ring dihedral angle, 3.46 (1)°] (Fig. 3).

### Experimental

The title compound was synthesized by heating together under reflux for 10 minutes, 1 mmol of zinc(II) iodide and 2 mmol of 4-(4-nitrobenzyl)pyridine in 50 ml of 50% ethanol–water. After concentration to *ca* 30 ml, partial room temperature evaporation of the hot-filtered solution gave pale yellow flattened prisms of (I) from which a suitable specimen was cleaved for the X-ray analysis.

### Refinement

Hydrogen atoms were included in the refinement in calculated positions with C–H = 0.93 Å (aromatic) or 0.97 Å (aliphatic) and allowed to ride, with *U*_iso_(H) = 1.2*U*_eq_(C). A racemic twin was identified and treated as such using the appropriate *SHELXL97* function [BASF factor, 0.430 (14)]. Oxygen atoms of the terminal nitro groups were significantly disordered, one in particular (O41*C*, *U*_iso_ = 0.142 Å^2^) subsequently being refined isotropically.

### Crystal data

**Table:**

[ZnI_2_(C_12_H_10_N_2_O_2_)_2_]	*D*_x_ = 1.802 Mg m^−^^3^
*M**_r_* = 747.61	Melting point = 473–475 K
Orthorhombic, *P**c**a*2_1_	Mo *K*α radiation, λ = 0.71073 Å
Hall symbol: P 2c -2ac	Cell parameters from 5528 reflections
*a* = 18.2091 (2) Å	θ = 3.3–28.7°
*b* = 15.8998 (3) Å	µ = 3.17 mm^−^^1^
*c* = 19.0327 (3) Å	*T* = 200 K
*V* = 5510.37 (15) Å^3^	Prism, pale yellow
*Z* = 8	0.40 × 0.22 × 0.15 mm
*F*(000) = 2880	

### Data collection

**Table:**

Oxford Diffraction Gemini-S Ultra CCD detector diffractometer	10102 independent reflections
Radiation source: Enhance (Mo) X-ray source	7499 reflections with *I* > 2σ(*I*)
graphite	*R*_int_ = 0.024
Detector resolution: 16.077 pixels mm^-1^	θ_max_ = 26.0°, θ_min_ = 3.4°
ω scans	*h* = −22→22
Absorption correction: multi-scan (*CrysAlis PRO*; Oxford Diffraction, 2010)	*k* = −19→19
*T*_min_ = 0.865, *T*_max_ = 0.980	*l* = −23→22
18536 measured reflections	

### Refinement

**Table:**

Refinement on *F*^2^	Secondary atom site location: difference Fourier map
Least-squares matrix: full	Hydrogen site location: inferred from neighbouring sites
*R*[*F*^2^ > 2σ(*F*^2^)] = 0.032	H-atom parameters not refined
*wR*(*F*^2^) = 0.056	*w* = 1/[σ^2^(*F*_o_^2^) + (0.0262*P*)^2^] where *P* = (*F*_o_^2^ + 2*F*_c_^2^)/3
*S* = 0.89	(Δ/σ)_max_ = 0.024
10102 reflections	Δρ_max_ = 0.63 e Å^−^^3^
627 parameters	Δρ_min_ = −0.47 e Å^−^^3^
25 restraints	Absolute structure: Flack (1983), 4516 Friedel pairs
Primary atom site location: structure-invariant direct methods	Flack parameter: 0.430 (14)

### Special details

**Table:**

Geometry. Bond distances, angles *etc*. have been calculated using the rounded fractional coordinates. All su's are estimated from the variances of the (full) variance-covariance matrix. The cell e.s.d.'s are taken into account in the estimation of distances, angles and torsion angles
Refinement. Refinement of *F*^2^ against ALL reflections. The weighted *R*-factor *wR* and goodness of fit *S* are based on *F*^2^, conventional *R*-factors *R* are based on *F*, with *F* set to zero for negative *F*^2^. The threshold expression of *F*^2^ > σ(*F*^2^) is used only for calculating *R*-factors(gt) *etc*. and is not relevant to the choice of reflections for refinement. *R*-factors based on *F*^2^ are statistically about twice as large as those based on *F*, and *R*- factors based on ALL data will be even larger.

### Fractional atomic coordinates and isotropic or equivalent isotropic displacement parameters (Å^2^)

**Table:**

	*x*	*y*	*z*	*U*_iso_*/*U*_eq_	
I1	0.95618 (2)	0.12807 (3)	0.69072 (2)	0.0518 (1)	
I2	1.15212 (2)	0.14231 (3)	0.82772 (2)	0.0567 (1)	
Zn1	1.01354 (3)	0.12528 (4)	0.81338 (3)	0.0432 (2)	
O41A	0.6840 (4)	0.3754 (6)	1.2662 (4)	0.179 (5)	
O41B	0.6256 (3)	−0.0954 (3)	1.1910 (3)	0.0767 (19)	
O42A	0.7998 (4)	0.3645 (5)	1.3010 (4)	0.123 (3)	
O42B	0.5740 (3)	−0.1013 (4)	1.0908 (3)	0.100 (3)	
N1A	0.9587 (2)	0.2149 (3)	0.8705 (2)	0.0440 (17)	
N1B	0.9820 (2)	0.0205 (3)	0.8686 (2)	0.0393 (16)	
N41A	0.7532 (6)	0.3685 (6)	1.2560 (5)	0.146 (5)	
N41B	0.6283 (3)	−0.1037 (3)	1.1280 (4)	0.060 (2)	
C2A	0.8847 (3)	0.2146 (4)	0.8703 (3)	0.064 (3)	
C2B	1.0121 (3)	0.0043 (3)	0.9320 (3)	0.0413 (19)	
C3A	0.8434 (3)	0.2746 (4)	0.9017 (3)	0.065 (3)	
C3B	0.9891 (3)	−0.0596 (4)	0.9745 (3)	0.0447 (19)	
C4A	0.8780 (4)	0.3398 (4)	0.9357 (3)	0.053 (2)	
C4B	0.9333 (3)	−0.1124 (3)	0.9535 (3)	0.038 (2)	
C5A	0.9519 (4)	0.3414 (4)	0.9358 (4)	0.066 (3)	
C5B	0.9039 (3)	−0.0982 (4)	0.8879 (3)	0.045 (2)	
C6A	0.9918 (3)	0.2776 (4)	0.9036 (3)	0.058 (3)	
C6B	0.9289 (3)	−0.0329 (3)	0.8476 (3)	0.0440 (19)	
C11A	0.8119 (4)	0.3939 (4)	1.0439 (4)	0.056 (3)	
C11B	0.8332 (3)	−0.1589 (3)	1.0348 (3)	0.0343 (19)	
C21A	0.7428 (4)	0.3758 (6)	1.0609 (5)	0.098 (4)	
C21B	0.7678 (3)	−0.1709 (3)	0.9985 (4)	0.049 (2)	
C31A	0.7239 (4)	0.3700 (6)	1.1283 (5)	0.107 (4)	
C31B	0.7012 (3)	−0.1499 (4)	1.0273 (3)	0.052 (2)	
C41A	0.7739 (4)	0.3750 (5)	1.1802 (5)	0.078 (3)	
C41B	0.7001 (3)	−0.1210 (3)	1.0951 (3)	0.040 (2)	
C42A	0.8329 (4)	0.4105 (4)	0.9687 (4)	0.080 (3)	
C42B	0.9056 (3)	−0.1818 (3)	1.0005 (3)	0.0453 (19)	
C51A	0.8454 (4)	0.3902 (4)	1.1653 (5)	0.072 (3)	
C51B	0.7630 (3)	−0.1092 (3)	1.1331 (3)	0.042 (2)	
C61A	0.8651 (4)	0.3999 (4)	1.0951 (4)	0.068 (3)	
C61B	0.8296 (3)	−0.1277 (4)	1.1026 (4)	0.048 (2)	
I3	1.16361 (2)	0.37425 (3)	0.36527 (2)	0.0519 (2)	
I4	0.97184 (2)	0.40341 (3)	0.50705 (2)	0.0539 (1)	
Zn2	1.02497 (3)	0.39353 (4)	0.38352 (4)	0.0426 (2)	
O41C	0.6595 (4)	0.1413 (4)	−0.0106 (4)	0.142 (3)*	
O41D	0.6107 (3)	0.6158 (3)	−0.0002 (4)	0.087 (3)	
O42C	0.7457 (4)	0.0960 (4)	−0.0805 (4)	0.111 (3)	
O42D	0.5606 (3)	0.6355 (4)	0.0996 (3)	0.100 (3)	
N1C	0.9779 (2)	0.2952 (3)	0.3310 (2)	0.0397 (16)	
N1D	0.9879 (2)	0.4909 (3)	0.3227 (2)	0.0410 (16)	
N41C	0.7239 (3)	0.1135 (5)	−0.0243 (4)	0.095 (3)	
N41D	0.6136 (3)	0.6286 (4)	0.0638 (4)	0.072 (3)	
C2C	0.9348 (3)	0.2378 (4)	0.3602 (3)	0.0467 (19)	
C2D	1.0279 (3)	0.5244 (4)	0.2716 (3)	0.060 (2)	
C3C	0.9099 (3)	0.1676 (4)	0.3250 (3)	0.050 (2)	
C3D	1.0007 (3)	0.5839 (4)	0.2253 (3)	0.056 (2)	
C4C	0.9291 (3)	0.1548 (4)	0.2565 (4)	0.048 (2)	
C4D	0.9294 (3)	0.6108 (3)	0.2306 (3)	0.0383 (19)	
C5C	0.9721 (3)	0.2143 (4)	0.2250 (3)	0.0510 (19)	
C5D	0.8890 (3)	0.5774 (4)	0.2850 (3)	0.055 (2)	
C6C	0.9953 (3)	0.2838 (4)	0.2631 (3)	0.054 (2)	
C6D	0.9198 (3)	0.5191 (3)	0.3297 (3)	0.053 (2)	
C11C	0.8593 (3)	0.0910 (3)	0.1544 (3)	0.0437 (19)	
C11D	0.8235 (3)	0.6580 (3)	0.1517 (3)	0.0400 (19)	
C21C	0.8846 (3)	0.0790 (4)	0.0877 (4)	0.056 (3)	
C21D	0.8159 (3)	0.6233 (3)	0.0871 (3)	0.040 (2)	
C31C	0.8426 (3)	0.0866 (4)	0.0299 (4)	0.060 (3)	
C31D	0.7475 (3)	0.6113 (3)	0.0565 (4)	0.049 (2)	
C41C	0.7692 (3)	0.1101 (4)	0.0397 (4)	0.058 (3)	
C41D	0.6878 (3)	0.6352 (4)	0.0956 (3)	0.044 (2)	
C42C	0.9073 (4)	0.0765 (4)	0.2176 (3)	0.061 (3)	
C42D	0.8996 (3)	0.6763 (4)	0.1819 (3)	0.049 (2)	
C51C	0.7415 (4)	0.1250 (5)	0.1052 (4)	0.085 (4)	
C51D	0.6927 (3)	0.6680 (4)	0.1617 (3)	0.050 (2)	
C61C	0.7878 (3)	0.1152 (4)	0.1627 (4)	0.069 (3)	
C61D	0.7615 (3)	0.6803 (3)	0.1900 (4)	0.051 (2)	
H2A	0.86070	0.17080	0.84750	0.0770*	
H2B	1.05040	0.03830	0.94750	0.0500*	
H3A	0.79250	0.27180	0.90020	0.0770*	
H3B	1.01130	−0.06760	1.01810	0.0540*	
H5A	0.97650	0.38550	0.95760	0.0800*	
H5B	0.86700	−0.13300	0.87090	0.0540*	
H6A	1.04280	0.27900	0.90520	0.0690*	
H6B	0.90830	−0.02500	0.80340	0.0530*	
H21A	0.70790	0.36740	1.02590	0.1180*	
H21B	0.76920	−0.19370	0.95350	0.0590*	
H31A	0.67470	0.36220	1.13990	0.1280*	
H31B	0.65800	−0.15520	1.00160	0.0630*	
H42A	0.86090	0.46230	0.96660	0.0960*	
H42B	0.94180	−0.19310	1.03670	0.0540*	
H43A	0.78850	0.41860	0.94130	0.0960*	
H43B	0.89930	−0.23270	0.97310	0.0540*	
H51A	0.88020	0.39410	1.20090	0.0860*	
H51B	0.76080	−0.08890	1.17890	0.0500*	
H61A	0.91370	0.41040	1.08290	0.0820*	
H61B	0.87270	−0.11920	1.12790	0.0570*	
H2C	0.92080	0.24520	0.40670	0.0560*	
H2D	1.07640	0.50710	0.26670	0.0720*	
H3C	0.87990	0.12900	0.34790	0.0600*	
H3D	1.03090	0.60580	0.19040	0.0670*	
H5C	0.98570	0.20820	0.17820	0.0610*	
H5D	0.84060	0.59430	0.29160	0.0660*	
H6	0.89140	0.49860	0.36650	0.0630*	
H6C	1.02390	0.32410	0.24070	0.0650*	
H21C	0.93370	0.06470	0.08190	0.0670*	
H21D	0.85770	0.60700	0.06250	0.0480*	
H31C	0.86160	0.07670	−0.01470	0.0720*	
H31D	0.74240	0.58810	0.01190	0.0580*	
H42C	0.88150	0.03960	0.24980	0.0730*	
H42D	0.93370	0.68300	0.14310	0.0590*	
H43C	0.95150	0.04760	0.20250	0.0730*	
H43D	0.89760	0.72940	0.20680	0.0590*	
H51C	0.69290	0.14130	0.11120	0.1020*	
H51D	0.65060	0.68170	0.18700	0.0600*	
H61C	0.76970	0.12520	0.20770	0.0830*	
H61D	0.76650	0.70340	0.23460	0.0610*	

### Atomic displacement parameters (Å^2^)

**Table:**

	*U*^11^	*U*^22^	*U*^33^	*U*^12^	*U*^13^	*U*^23^
I1	0.0442 (2)	0.0799 (3)	0.0313 (2)	−0.0069 (2)	−0.0057 (2)	0.0026 (2)
I2	0.0398 (2)	0.0944 (3)	0.0359 (2)	−0.0126 (2)	−0.0025 (2)	0.0057 (2)
Zn1	0.0402 (3)	0.0605 (4)	0.0289 (4)	−0.0065 (3)	−0.0014 (3)	0.0019 (3)
O41A	0.108 (6)	0.285 (11)	0.143 (8)	0.053 (6)	0.055 (5)	0.074 (6)
O41B	0.081 (3)	0.107 (4)	0.042 (3)	0.013 (3)	0.015 (3)	−0.013 (3)
O42A	0.097 (4)	0.186 (6)	0.085 (5)	0.013 (5)	0.010 (4)	0.029 (5)
O42B	0.048 (3)	0.181 (6)	0.070 (4)	0.016 (3)	0.000 (3)	−0.004 (4)
N1A	0.046 (3)	0.055 (3)	0.031 (3)	−0.008 (2)	−0.001 (2)	−0.005 (2)
N1B	0.036 (2)	0.054 (3)	0.028 (3)	−0.002 (2)	−0.001 (2)	0.003 (2)
N41A	0.100 (6)	0.260 (12)	0.079 (7)	0.001 (8)	0.027 (5)	0.046 (8)
N41B	0.068 (4)	0.062 (4)	0.051 (4)	0.005 (3)	0.011 (3)	0.002 (3)
C2A	0.061 (4)	0.063 (4)	0.068 (5)	−0.004 (3)	0.005 (4)	−0.009 (4)
C2B	0.036 (3)	0.058 (4)	0.030 (3)	−0.006 (3)	−0.001 (3)	0.000 (3)
C3A	0.057 (4)	0.070 (5)	0.067 (5)	0.009 (4)	−0.003 (3)	−0.007 (4)
C3B	0.036 (3)	0.070 (4)	0.028 (3)	0.004 (3)	−0.001 (3)	0.002 (3)
C4A	0.062 (4)	0.056 (4)	0.041 (4)	0.013 (4)	0.010 (3)	0.005 (3)
C4B	0.036 (3)	0.047 (4)	0.032 (4)	0.007 (3)	0.006 (3)	−0.004 (3)
C5A	0.084 (5)	0.066 (5)	0.049 (4)	−0.013 (4)	−0.002 (4)	−0.006 (4)
C5B	0.043 (3)	0.050 (4)	0.041 (4)	−0.008 (3)	−0.008 (3)	−0.002 (3)
C6A	0.057 (4)	0.072 (5)	0.045 (4)	−0.001 (4)	−0.003 (3)	−0.003 (3)
C6B	0.046 (3)	0.059 (4)	0.027 (3)	−0.004 (3)	−0.009 (2)	0.002 (3)
C11A	0.063 (4)	0.055 (4)	0.049 (5)	0.023 (3)	−0.007 (4)	−0.008 (3)
C11B	0.038 (3)	0.031 (3)	0.034 (4)	0.003 (3)	0.006 (2)	0.002 (3)
C21A	0.048 (4)	0.168 (9)	0.079 (7)	0.006 (5)	−0.014 (4)	−0.020 (6)
C21B	0.044 (3)	0.064 (4)	0.039 (4)	0.004 (3)	0.001 (3)	−0.007 (3)
C31A	0.056 (5)	0.196 (10)	0.068 (6)	−0.019 (6)	0.003 (4)	0.014 (7)
C31B	0.044 (3)	0.066 (4)	0.046 (4)	0.003 (3)	−0.013 (3)	−0.001 (3)
C41A	0.063 (4)	0.108 (6)	0.064 (6)	0.008 (4)	0.005 (4)	0.004 (5)
C41B	0.041 (3)	0.036 (4)	0.042 (4)	0.007 (3)	0.008 (3)	0.005 (3)
C42A	0.090 (5)	0.062 (5)	0.089 (6)	0.034 (4)	−0.017 (5)	−0.002 (4)
C42B	0.045 (3)	0.041 (3)	0.050 (4)	0.003 (3)	−0.004 (3)	0.006 (3)
C51A	0.050 (4)	0.096 (6)	0.069 (6)	0.012 (4)	−0.003 (4)	0.000 (4)
C51B	0.059 (4)	0.039 (4)	0.027 (4)	0.005 (3)	0.007 (3)	0.005 (3)
C61A	0.049 (4)	0.080 (5)	0.076 (6)	0.004 (4)	0.000 (4)	0.000 (4)
C61B	0.046 (3)	0.053 (4)	0.045 (4)	−0.006 (3)	−0.005 (3)	0.006 (3)
I3	0.0382 (2)	0.0769 (3)	0.0405 (3)	0.0048 (2)	−0.0001 (2)	−0.0048 (2)
I4	0.0483 (2)	0.0798 (3)	0.0335 (2)	0.0107 (2)	0.0042 (2)	0.0021 (2)
Zn2	0.0391 (3)	0.0570 (5)	0.0317 (4)	0.0012 (3)	−0.0014 (3)	0.0019 (3)
O41D	0.075 (3)	0.112 (5)	0.075 (5)	−0.010 (3)	−0.030 (3)	0.002 (3)
O42C	0.101 (4)	0.171 (6)	0.062 (5)	−0.020 (4)	−0.020 (4)	0.002 (4)
O42D	0.045 (3)	0.154 (5)	0.100 (5)	0.004 (3)	0.000 (3)	−0.028 (4)
N1C	0.035 (2)	0.049 (3)	0.035 (3)	−0.003 (2)	0.003 (2)	−0.001 (2)
N1D	0.038 (2)	0.056 (3)	0.029 (3)	0.001 (2)	0.001 (2)	0.003 (2)
N41C	0.049 (4)	0.158 (7)	0.077 (6)	−0.013 (4)	−0.012 (4)	0.017 (5)
N41D	0.057 (4)	0.086 (5)	0.073 (5)	−0.001 (3)	−0.016 (4)	0.001 (4)
C2C	0.044 (3)	0.062 (4)	0.034 (3)	0.007 (3)	0.003 (3)	−0.001 (3)
C2D	0.035 (3)	0.086 (5)	0.060 (4)	0.013 (3)	0.006 (3)	0.019 (4)
C3C	0.050 (3)	0.056 (4)	0.044 (4)	−0.008 (3)	0.003 (3)	0.011 (3)
C3D	0.037 (3)	0.081 (5)	0.050 (4)	−0.002 (3)	0.011 (3)	0.031 (4)
C4C	0.045 (3)	0.044 (4)	0.055 (5)	0.002 (3)	−0.018 (3)	0.008 (3)
C4D	0.047 (3)	0.040 (4)	0.028 (3)	−0.007 (3)	−0.004 (3)	0.002 (3)
C5C	0.055 (3)	0.065 (4)	0.033 (3)	−0.003 (3)	0.003 (3)	−0.004 (3)
C5D	0.043 (3)	0.074 (5)	0.047 (4)	0.011 (3)	0.010 (3)	0.020 (4)
C6C	0.045 (3)	0.074 (5)	0.043 (4)	−0.010 (3)	−0.003 (3)	0.007 (3)
C6D	0.046 (3)	0.069 (4)	0.043 (4)	−0.003 (3)	0.003 (3)	0.012 (3)
C11C	0.044 (3)	0.035 (3)	0.052 (4)	−0.004 (3)	−0.006 (3)	−0.007 (3)
C11D	0.043 (3)	0.038 (3)	0.039 (4)	−0.010 (3)	−0.002 (3)	0.017 (3)
C21C	0.048 (3)	0.066 (5)	0.054 (5)	0.006 (3)	0.003 (3)	−0.005 (4)
C21D	0.051 (3)	0.039 (4)	0.030 (4)	0.002 (3)	0.006 (3)	−0.001 (3)
C31C	0.051 (4)	0.072 (5)	0.056 (5)	−0.003 (3)	0.009 (3)	−0.001 (4)
C31D	0.054 (3)	0.054 (4)	0.038 (4)	0.006 (3)	−0.007 (3)	−0.003 (3)
C41C	0.045 (3)	0.082 (5)	0.048 (5)	−0.016 (3)	−0.015 (3)	0.006 (4)
C41D	0.043 (3)	0.046 (4)	0.042 (4)	0.005 (3)	−0.009 (3)	−0.002 (3)
C42C	0.083 (5)	0.050 (4)	0.049 (4)	0.001 (3)	−0.011 (4)	−0.009 (3)
C42D	0.047 (3)	0.062 (4)	0.038 (4)	−0.002 (3)	0.002 (3)	0.002 (3)
C51C	0.042 (4)	0.160 (8)	0.054 (6)	0.012 (4)	0.007 (4)	−0.003 (5)
C51D	0.040 (3)	0.060 (4)	0.050 (4)	0.004 (3)	0.006 (3)	0.002 (3)
C61C	0.053 (4)	0.107 (6)	0.048 (5)	0.004 (4)	0.014 (3)	−0.011 (4)
C61D	0.057 (4)	0.064 (4)	0.031 (3)	−0.004 (3)	0.006 (3)	−0.004 (3)

### Geometric parameters (Å, °)

**Table:**

I1—Zn1	2.5578 (7)	C6A—H6A	0.9300
I2—Zn1	2.5525 (7)	C6B—H6B	0.9300
I3—Zn2	2.5666 (7)	C21A—H21A	0.9300
I4—Zn2	2.5472 (8)	C21B—H21B	0.9300
Zn1—N1A	2.052 (4)	C31A—H31A	0.9300
Zn1—N1B	2.052 (4)	C31B—H31B	0.9300
Zn2—N1C	2.044 (4)	C42A—H42A	0.9700
Zn2—N1D	2.048 (4)	C42A—H43A	0.9700
O41A—N41A	1.280 (13)	C42B—H42B	0.9700
O41B—N41B	1.207 (9)	C42B—H43B	0.9700
O42A—N41A	1.207 (13)	C51A—H51A	0.9300
O42B—N41B	1.217 (8)	C51B—H51B	0.9300
O41C—N41C	1.280 (9)	C61A—H61A	0.9300
O41D—N41D	1.236 (11)	C61B—H61B	0.9300
O42C—N41C	1.174 (11)	C2C—C3C	1.379 (9)
O42D—N41D	1.187 (8)	C2D—C3D	1.385 (8)
N1A—C2A	1.348 (7)	C3C—C4C	1.365 (9)
N1A—C6A	1.324 (7)	C3D—C4D	1.371 (8)
N1B—C2B	1.350 (7)	C4C—C5C	1.367 (9)
N1B—C6B	1.347 (7)	C4C—C42C	1.502 (9)
N41A—C41A	1.495 (13)	C4D—C5D	1.377 (8)
N41B—C41B	1.476 (8)	C4D—C42D	1.496 (8)
N1C—C6C	1.343 (7)	C5C—C6C	1.388 (9)
N1C—C2C	1.326 (7)	C5D—C6D	1.378 (8)
N1D—C6D	1.325 (7)	C11C—C21C	1.364 (9)
N1D—C2D	1.327 (7)	C11C—C42C	1.505 (8)
N41C—C41C	1.472 (10)	C11C—C61C	1.367 (8)
N41D—C41D	1.484 (8)	C11D—C21D	1.355 (8)
C2A—C3A	1.354 (8)	C11D—C42D	1.528 (8)
C2B—C3B	1.365 (8)	C11D—C61D	1.390 (8)
C3A—C4A	1.375 (9)	C21C—C31C	1.345 (10)
C3B—C4B	1.377 (8)	C21D—C31D	1.388 (8)
C4A—C5A	1.346 (10)	C31C—C41C	1.400 (8)
C4A—C42A	1.527 (9)	C31D—C41D	1.371 (8)
C4B—C42B	1.507 (7)	C41C—C51C	1.366 (11)
C4B—C5B	1.377 (8)	C41D—C51D	1.365 (8)
C5A—C6A	1.390 (9)	C51C—C61C	1.390 (10)
C5B—C6B	1.369 (8)	C51D—C61D	1.378 (8)
C11A—C61A	1.377 (11)	C2C—H2C	0.9300
C11A—C21A	1.331 (10)	C2D—H2D	0.9300
C11A—C42A	1.505 (11)	C3C—H3C	0.9300
C11B—C42B	1.516 (8)	C3D—H3D	0.9300
C11B—C61B	1.384 (9)	C5C—H5C	0.9300
C11B—C21B	1.390 (8)	C5D—H5D	0.9300
C21A—C31A	1.331 (13)	C6C—H6C	0.9300
C21B—C31B	1.372 (8)	C6D—H6	0.9300
C31A—C41A	1.346 (12)	C21C—H21C	0.9300
C31B—C41B	1.370 (8)	C21D—H21D	0.9300
C41A—C51A	1.354 (10)	C31C—H31C	0.9300
C41B—C51B	1.368 (8)	C31D—H31D	0.9300
C51A—C61A	1.392 (12)	C42C—H42C	0.9700
C51B—C61B	1.376 (8)	C42C—H43C	0.9700
C2A—H2A	0.9300	C42D—H42D	0.9700
C2B—H2B	0.9300	C42D—H43D	0.9700
C3A—H3A	0.9300	C51C—H51C	0.9300
C3B—H3B	0.9300	C51D—H51D	0.9300
C5A—H5A	0.9300	C61C—H61C	0.9300
C5B—H5B	0.9300	C61D—H61D	0.9300
			
I1—Zn1—I2	119.96 (2)	C4A—C42A—H43A	109.00
I1—Zn1—N1A	105.83 (11)	C4A—C42A—H42A	109.00
I1—Zn1—N1B	111.55 (11)	H42A—C42A—H43A	108.00
I2—Zn1—N1A	110.54 (11)	C11A—C42A—H43A	109.00
I2—Zn1—N1B	107.94 (11)	C11A—C42A—H42A	109.00
N1A—Zn1—N1B	98.99 (17)	C11B—C42B—H42B	109.00
I4—Zn2—N1D	110.49 (11)	C4B—C42B—H43B	109.00
N1C—Zn2—N1D	99.42 (17)	C4B—C42B—H42B	109.00
I3—Zn2—I4	120.39 (3)	C11B—C42B—H43B	109.00
I3—Zn2—N1C	104.77 (11)	H42B—C42B—H43B	108.00
I3—Zn2—N1D	109.76 (11)	C41A—C51A—H51A	121.00
I4—Zn2—N1C	109.83 (11)	C61A—C51A—H51A	121.00
Zn1—N1A—C6A	123.6 (3)	C61B—C51B—H51B	120.00
C2A—N1A—C6A	117.3 (5)	C41B—C51B—H51B	120.00
Zn1—N1A—C2A	118.9 (4)	C11A—C61A—H61A	120.00
Zn1—N1B—C6B	124.1 (3)	C51A—C61A—H61A	120.00
Zn1—N1B—C2B	119.9 (3)	C11B—C61B—H61B	120.00
C2B—N1B—C6B	115.9 (4)	C51B—C61B—H61B	120.00
O41A—N41A—C41A	112.9 (8)	N1C—C2C—C3C	123.3 (5)
O41A—N41A—O42A	126.1 (9)	N1D—C2D—C3D	123.0 (5)
O42A—N41A—C41A	120.7 (9)	C2C—C3C—C4C	120.0 (6)
O42B—N41B—C41B	118.6 (7)	C2D—C3D—C4D	120.4 (5)
O41B—N41B—O42B	122.8 (6)	C3C—C4C—C5C	117.6 (6)
O41B—N41B—C41B	118.6 (6)	C3C—C4C—C42C	121.8 (6)
Zn2—N1C—C2C	124.7 (4)	C5C—C4C—C42C	120.6 (6)
Zn2—N1C—C6C	118.4 (4)	C3D—C4D—C5D	116.2 (5)
C2C—N1C—C6C	116.8 (5)	C3D—C4D—C42D	121.0 (5)
Zn2—N1D—C2D	122.5 (4)	C5D—C4D—C42D	122.7 (5)
Zn2—N1D—C6D	120.5 (3)	C4C—C5C—C6C	119.8 (6)
C2D—N1D—C6D	116.8 (5)	C4D—C5D—C6D	120.4 (5)
O41C—N41C—O42C	125.2 (7)	N1C—C6C—C5C	122.6 (5)
O41C—N41C—C41C	110.9 (7)	N1D—C6D—C5D	123.1 (5)
O42C—N41C—C41C	123.8 (6)	C21C—C11C—C42C	121.8 (5)
O41D—N41D—C41D	116.9 (6)	C21C—C11C—C61C	118.0 (6)
O42D—N41D—C41D	120.0 (7)	C42C—C11C—C61C	120.3 (6)
O41D—N41D—O42D	123.1 (6)	C21D—C11D—C42D	120.8 (5)
N1A—C2A—C3A	123.5 (5)	C21D—C11D—C61D	119.8 (5)
N1B—C2B—C3B	123.2 (5)	C42D—C11D—C61D	119.4 (5)
C2A—C3A—C4A	119.0 (5)	C11C—C21C—C31C	123.8 (5)
C2B—C3B—C4B	120.6 (5)	C11D—C21D—C31D	121.9 (5)
C3A—C4A—C5A	118.2 (6)	C21C—C31C—C41C	117.2 (7)
C3A—C4A—C42A	120.1 (6)	C21D—C31D—C41D	116.5 (6)
C5A—C4A—C42A	121.5 (6)	N41C—C41C—C31C	115.8 (6)
C3B—C4B—C5B	116.7 (5)	N41C—C41C—C51C	122.8 (6)
C3B—C4B—C42B	121.4 (5)	C31C—C41C—C51C	121.4 (7)
C5B—C4B—C42B	121.9 (5)	N41D—C41D—C31D	118.7 (6)
C4A—C5A—C6A	120.5 (6)	N41D—C41D—C51D	117.5 (5)
C4B—C5B—C6B	120.2 (5)	C31D—C41D—C51D	123.7 (5)
N1A—C6A—C5A	121.4 (5)	C4C—C42C—C11C	114.9 (5)
N1B—C6B—C5B	123.4 (5)	C4D—C42D—C11D	115.4 (5)
C21A—C11A—C42A	120.6 (7)	C41C—C51C—C61C	118.4 (6)
C42A—C11A—C61A	118.8 (6)	C41D—C51D—C61D	118.3 (5)
C21A—C11A—C61A	120.5 (8)	C11C—C61C—C51C	121.2 (7)
C21B—C11B—C61B	118.2 (5)	C11D—C61D—C51D	119.8 (6)
C42B—C11B—C61B	121.9 (5)	N1C—C2C—H2C	118.00
C21B—C11B—C42B	119.9 (5)	C3C—C2C—H2C	118.00
C11A—C21A—C31A	119.6 (8)	N1D—C2D—H2D	118.00
C11B—C21B—C31B	121.7 (6)	C3D—C2D—H2D	119.00
C21A—C31A—C41A	121.9 (7)	C2C—C3C—H3C	120.00
C21B—C31B—C41B	118.1 (5)	C4C—C3C—H3C	120.00
N41A—C41A—C51A	117.2 (8)	C2D—C3D—H3D	120.00
C31A—C41A—C51A	120.5 (9)	C4D—C3D—H3D	120.00
N41A—C41A—C31A	122.3 (8)	C4C—C5C—H5C	120.00
N41B—C41B—C51B	119.5 (5)	C6C—C5C—H5C	120.00
C31B—C41B—C51B	122.1 (5)	C4D—C5D—H5D	120.00
N41B—C41B—C31B	118.4 (5)	C6D—C5D—H5D	120.00
C4A—C42A—C11A	113.5 (5)	N1C—C6C—H6C	119.00
C4B—C42B—C11B	111.8 (4)	C5C—C6C—H6C	119.00
C41A—C51A—C61A	117.9 (8)	N1D—C6D—H6	119.00
C41B—C51B—C61B	119.1 (6)	C5D—C6D—H6	118.00
C11A—C61A—C51A	119.4 (7)	C11C—C21C—H21C	118.00
C11B—C61B—C51B	120.8 (5)	C31C—C21C—H21C	118.00
N1A—C2A—H2A	118.00	C11D—C21D—H21D	119.00
C3A—C2A—H2A	118.00	C31D—C21D—H21D	119.00
N1B—C2B—H2B	118.00	C21C—C31C—H31C	121.00
C3B—C2B—H2B	118.00	C41C—C31C—H31C	121.00
C2A—C3A—H3A	120.00	C21D—C31D—H31D	122.00
C4A—C3A—H3A	121.00	C41D—C31D—H31D	122.00
C4B—C3B—H3B	120.00	C4C—C42C—H42C	109.00
C2B—C3B—H3B	120.00	C4C—C42C—H43C	109.00
C4A—C5A—H5A	120.00	C11C—C42C—H42C	108.00
C6A—C5A—H5A	120.00	C11C—C42C—H43C	109.00
C4B—C5B—H5B	120.00	H42C—C42C—H43C	108.00
C6B—C5B—H5B	120.00	C4D—C42D—H42D	108.00
N1A—C6A—H6A	119.00	C4D—C42D—H43D	108.00
C5A—C6A—H6A	119.00	C11D—C42D—H42D	108.00
C5B—C6B—H6B	118.00	C11D—C42D—H43D	108.00
N1B—C6B—H6B	118.00	H42D—C42D—H43D	107.00
C11A—C21A—H21A	120.00	C41C—C51C—H51C	121.00
C31A—C21A—H21A	120.00	C61C—C51C—H51C	121.00
C11B—C21B—H21B	119.00	C41D—C51D—H51D	121.00
C31B—C21B—H21B	119.00	C61D—C51D—H51D	121.00
C41A—C31A—H31A	119.00	C11C—C61C—H61C	119.00
C21A—C31A—H31A	119.00	C51C—C61C—H61C	119.00
C21B—C31B—H31B	121.00	C11D—C61D—H61D	120.00
C41B—C31B—H31B	121.00	C51D—C61D—H61D	120.00
			
I1—Zn1—N1A—C2A	49.9 (4)	C5B—C4B—C42B—C11B	−76.6 (7)
I1—Zn1—N1A—C6A	−124.7 (4)	C3B—C4B—C42B—C11B	102.9 (6)
I2—Zn1—N1A—C2A	−178.7 (4)	C4A—C5A—C6A—N1A	1.8 (10)
I2—Zn1—N1A—C6A	6.7 (5)	C4B—C5B—C6B—N1B	−0.3 (9)
N1B—Zn1—N1A—C2A	−65.6 (4)	C42A—C11A—C61A—C51A	175.8 (6)
N1B—Zn1—N1A—C6A	119.8 (4)	C61A—C11A—C21A—C31A	5.3 (13)
I1—Zn1—N1B—C2B	173.9 (3)	C21A—C11A—C42A—C4A	−108.2 (8)
I1—Zn1—N1B—C6B	−9.9 (4)	C42A—C11A—C21A—C31A	−173.0 (8)
I2—Zn1—N1B—C2B	40.1 (4)	C21A—C11A—C61A—C51A	−2.6 (10)
I2—Zn1—N1B—C6B	−143.7 (4)	C61A—C11A—C42A—C4A	73.5 (8)
N1A—Zn1—N1B—C2B	−75.0 (4)	C61B—C11B—C21B—C31B	2.3 (8)
N1A—Zn1—N1B—C6B	101.1 (4)	C42B—C11B—C21B—C31B	−179.1 (5)
I3—Zn2—N1D—C6D	−171.8 (4)	C21B—C11B—C42B—C4B	83.4 (6)
I4—Zn2—N1D—C2D	148.3 (4)	C61B—C11B—C42B—C4B	−98.1 (6)
I4—Zn2—N1D—C6D	−36.8 (4)	C21B—C11B—C61B—C51B	0.1 (8)
N1C—Zn2—N1D—C2D	−96.3 (4)	C42B—C11B—C61B—C51B	−178.5 (5)
N1C—Zn2—N1D—C6D	78.6 (4)	C11A—C21A—C31A—C41A	−5.6 (14)
I4—Zn2—N1C—C2C	−9.4 (5)	C11B—C21B—C31B—C41B	−3.8 (9)
I4—Zn2—N1C—C6C	175.3 (4)	C21A—C31A—C41A—N41A	−180.0 (9)
N1D—Zn2—N1C—C2C	−125.4 (4)	C21A—C31A—C41A—C51A	3.1 (14)
N1D—Zn2—N1C—C6C	59.4 (4)	C21B—C31B—C41B—N41B	−176.0 (5)
I3—Zn2—N1D—C2D	13.3 (5)	C21B—C31B—C41B—C51B	2.9 (8)
I3—Zn2—N1C—C2C	121.2 (4)	C31A—C41A—C51A—C61A	−0.3 (11)
I3—Zn2—N1C—C6C	−54.1 (4)	N41A—C41A—C51A—C61A	−177.4 (7)
C2A—N1A—C6A—C5A	−1.1 (8)	N41B—C41B—C51B—C61B	178.2 (5)
Zn1—N1A—C6A—C5A	173.6 (5)	C31B—C41B—C51B—C61B	−0.7 (8)
Zn1—N1A—C2A—C3A	−174.7 (5)	C41A—C51A—C61A—C11A	0.1 (10)
C6A—N1A—C2A—C3A	0.2 (8)	C41B—C51B—C61B—C11B	−0.9 (9)
Zn1—N1B—C6B—C5B	−173.9 (4)	N1C—C2C—C3C—C4C	−0.4 (9)
C2B—N1B—C6B—C5B	2.4 (8)	N1D—C2D—C3D—C4D	−0.3 (9)
Zn1—N1B—C2B—C3B	173.8 (4)	C2C—C3C—C4C—C5C	−1.1 (9)
C6B—N1B—C2B—C3B	−2.7 (8)	C2C—C3C—C4C—C42C	176.0 (6)
O41A—N41A—C41A—C51A	163.4 (8)	C2D—C3D—C4D—C5D	2.0 (8)
O41A—N41A—C41A—C31A	−13.6 (13)	C2D—C3D—C4D—C42D	178.8 (5)
O42A—N41A—C41A—C31A	172.3 (9)	C3C—C4C—C5C—C6C	0.9 (9)
O42A—N41A—C41A—C51A	−10.7 (13)	C42C—C4C—C5C—C6C	−176.2 (6)
O41B—N41B—C41B—C31B	164.8 (5)	C3C—C4C—C42C—C11C	118.7 (6)
O42B—N41B—C41B—C31B	−12.7 (8)	C5C—C4C—C42C—C11C	−64.4 (8)
O42B—N41B—C41B—C51B	168.3 (5)	C3D—C4D—C5D—C6D	−1.3 (8)
O41B—N41B—C41B—C51B	−14.2 (7)	C42D—C4D—C5D—C6D	−178.0 (5)
Zn2—N1C—C6C—C5C	173.4 (4)	C3D—C4D—C42D—C11D	135.3 (6)
Zn2—N1C—C2C—C3C	−173.3 (4)	C5D—C4D—C42D—C11D	−48.1 (8)
C6C—N1C—C2C—C3C	2.1 (8)	C4C—C5C—C6C—N1C	0.8 (9)
C2C—N1C—C6C—C5C	−2.2 (8)	C4D—C5D—C6D—N1D	−1.3 (9)
Zn2—N1D—C2D—C3D	172.8 (4)	C42C—C11C—C21C—C31C	176.1 (6)
C6D—N1D—C2D—C3D	−2.3 (8)	C61C—C11C—C21C—C31C	−2.5 (9)
C2D—N1D—C6D—C5D	3.1 (8)	C21C—C11C—C42C—C4C	110.0 (6)
Zn2—N1D—C6D—C5D	−172.2 (4)	C61C—C11C—C42C—C4C	−71.4 (7)
O42C—N41C—C41C—C31C	1.9 (11)	C21C—C11C—C61C—C51C	1.8 (9)
O42C—N41C—C41C—C51C	−175.3 (8)	C42C—C11C—C61C—C51C	−176.9 (6)
O41C—N41C—C41C—C31C	−175.3 (6)	C42D—C11D—C21D—C31D	−175.6 (5)
O41C—N41C—C41C—C51C	7.5 (10)	C61D—C11D—C21D—C31D	1.9 (8)
O41D—N41D—C41D—C51D	165.3 (6)	C21D—C11D—C42D—C4D	−97.1 (6)
O42D—N41D—C41D—C31D	167.7 (6)	C61D—C11D—C42D—C4D	85.4 (6)
O42D—N41D—C41D—C51D	−14.8 (10)	C21D—C11D—C61D—C51D	−0.8 (8)
O41D—N41D—C41D—C31D	−12.3 (9)	C42D—C11D—C61D—C51D	176.7 (5)
N1A—C2A—C3A—C4A	0.1 (9)	C11C—C21C—C31C—C41C	1.5 (10)
N1B—C2B—C3B—C4B	0.9 (9)	C11D—C21D—C31D—C41D	−1.0 (8)
C2A—C3A—C4A—C42A	177.2 (6)	C21C—C31C—C41C—N41C	−176.9 (6)
C2A—C3A—C4A—C5A	0.5 (9)	C21C—C31C—C41C—C51C	0.3 (10)
C2B—C3B—C4B—C42B	−178.2 (5)	C21D—C31D—C41D—N41D	176.4 (5)
C2B—C3B—C4B—C5B	1.3 (8)	C21D—C31D—C41D—C51D	−1.1 (9)
C3A—C4A—C42A—C11A	89.9 (7)	N41C—C41C—C51C—C61C	176.1 (7)
C42A—C4A—C5A—C6A	−178.1 (6)	C31C—C41C—C51C—C61C	−0.9 (11)
C3A—C4A—C5A—C6A	−1.4 (10)	N41D—C41D—C51D—C61D	−175.3 (6)
C5A—C4A—C42A—C11A	−93.5 (8)	C31D—C41D—C51D—C61D	2.1 (10)
C3B—C4B—C5B—C6B	−1.6 (8)	C41C—C51C—C61C—C11C	−0.2 (10)
C42B—C4B—C5B—C6B	177.9 (5)	C41D—C51D—C61D—C11D	−1.1 (9)

### Hydrogen-bond geometry (Å, °)

**Table:**

*D*—H···*A*	*D*—H	H···*A*	*D*···*A*	*D*—H···*A*
C5A—H5A···O41D^i^	0.93	2.57	3.211 (9)	126
C6B—H6B···O41B^ii^	0.93	2.49	3.295 (8)	145

Symmetry codes: (i) *x*+1/2, −*y*+1, *z*+1; (ii) −*x*+3/2, *y*, *z*−1/2.
